# Design of urban innovation space system using artificial intelligence technology and internet of things

**DOI:** 10.1016/j.heliyon.2024.e25396

**Published:** 2024-01-27

**Authors:** Yifang Gao

**Affiliations:** College of Design and Innovation, Tongji University, Shanghai, 200092, China

**Keywords:** Artificial intelligence, Internet of things, Urban innovation space system, Visualization, Internet worm

## Abstract

The goal of this paper is to integrate artificial intelligence (AI) and Internet of things (IoT) technology into urban innovation space systems while expediting the construction of urban informatization. The core of the paper is to build an innovation space system, which is developed around three key components: innovation elements, innovation networks and innovation bases. First, the definition of innovation space is investigated in detail, and the essence of innovation space is understood to ensure that the key elements in the innovation process can be accurately captured and analyzed in the follow-up research. Second, it is clear that Chengdu is a representative city in Sichuan Province. Through the research in this area, people can deeply understand the specific background and characteristics of urban innovation space system. Then, the innovation space system is constructed, which is supported by innovation elements, innovation networks and innovation bases. These three components are intertwined, which together constitute the key elements of urban innovation space. Furthermore, the Internet worm technology is integrated with the IoT technology, and the system is visually inspected with the help of AI. The application of IoT technology helps to realize the automation and information sharing of the system, while the use of AI provides a deep insight into the system structure and operation. Through this research process, people can fully understand the construction process of Chengdu innovation space system, and provide deeper insight and support for urban innovation through the application of IoT and AI technology. The results show that while Chengdu's entrepreneurship and innovation enterprises are dispersed throughout all of the city's districts and counties, the city's academic talent and the bulk of its higher education institutions are concentrated in the city's core. There are 275 entrepreneurship and innovation enterprises in the High-tech District of Chengdu, which is the most densely distributed area. An urban innovation space network is being built by eight distinct research and higher education establishments. As urban innovation spaces are being built, emphasis should be given to the regional aggregation features of talents, higher education and research institutions, as well as entrepreneurship and innovation business enterprises. The innovation space system based on Internet worm technology of the IoT shows excellent performance in real-time identification of innovation elements, network connection quality, sensor monitoring, AI visual monitoring and so on. The system performs well in real-time monitoring of new enterprises and projects, and the real-time recognition rate reaches 98 %. The communication quality of the innovation network is relatively stable, and the connection quality reaches 92 %. The accuracy of sensor status monitoring in the IoT is high, reaching 99 %. The coverage of AI vision monitoring system reaches 96 %, effectively monitoring the areas involved in innovative space systems. Generally speaking, through the combination of theory and practice, this paaper provides comprehensive and specific guidance for the construction of urban innovation space system, promotes the research progress in this field, and makes beneficial contributions to the sustainable development of urban innovation and informatization.

## 1 Introduction

With the rapid development of science and technology and society, cities have become the engines of global innovation and development. In this emerging urban ecosystem, innovation space has gradually emerged and become the core factor to promote innovation and entrepreneurship [1]. Innovation space is not only the layout of buildings in the city, but also involves the construction of innovation networks and innovation bases [2]. This new urban structure aims to stimulate innovation, cultivate entrepreneurial spirit, and provide a platform for cooperation and exchange for innovators in different fields [3,4]. However, with the increasing attention of innovation space, how to make full use of artificial intelligence (AI) and Internet of Things (IoT) technologies and how to deeply understand the role of innovation space in urban development has become an urgent problem to be further studied [5].

The rise of AI and IoT technology can not only build smarter innovation space in cities, but also provide more efficient and convenient services [6,7]. For example, analyzing urban data through AI algorithm can better understand residents' needs, and the wide application of IoT devices makes it possible to collect real-time data, thus optimizing the design and operation of innovation space [8,9]. Innovation space is no longer just the physical structure of buildings, but also a multi-level and multi-factor system. In addition to the layout of physical space, the construction of innovation network and the development of innovation base have also become the key components of urban innovation ecology [10]. Through in-depth study of the relationship between these elements, people can better grasp the impact of innovation space on the overall situation of the city. The research should also focus on better understanding the specific role of innovation space in urban development. Innovation space is not only an incubator for startups, but also an engine to promote social and economic progress. This involves a deep understanding of the impact of innovation space on talent attraction, industrial agglomeration and urban competitiveness, thus providing more accurate guidance for urban planning. Currently, scholars cannot agree upon a concept of innovation space. While researchers in China and other countries have found that regional innovation elements are dominated by higher education institutions, industries, and governments, with intermediate enterprises serving as the foundation of regional innovation. Higher education institutions and governments actively promote industrial innovations and support enterprise innovation activities. The Chinese scholar Qi et al. (2019) [11] believed that innovation space should be classified according to different functions, including knowledge-based innovation space of basic research and industrial innovation space of high-tech industries. In short, although researchers have carefully studied the innovation space from different perspectives, such as origin, type, scale, definition, and structure, they have not formed a unified understanding of the innovation space theoretically, and the application of AI in the construction of innovation space system is rare.

Based on the above analysis, firstly, through a comprehensive definition of innovation space, this paper deeply excavates the key elements in the process of innovation, and ensures an accurate understanding of the essence of urban innovation space system. Secondly, Chengdu is chosen as the research site, and its representativeness in Sichuan Province is fully considered. Through the study of Chengdu's innovation space system, the local characteristics of urban innovation network are deeply understood. Thirdly, an innovation space system based on innovation elements, innovation networks and innovation bases is constructed. Through the construction of this system, the integration of key elements of urban innovation ecosystem has been realized, which provides an effective means for the coordinated promotion of innovation activities and the construction of industrial networks. In addition, the Internet worm technology is integrated by the IoT technology, and the system is visually inspected by AI, which injects advanced scientific and technological means into the urban innovation space system. The innovative application of this technology integration improves the automation degree and information sharing efficiency of the system, and provides in-depth insight into the system structure and operation through visual inspection, further enhancing the intelligence of the system. Finally, the numerical analysis results prove that the innovation space system based on Internet worm technology of IoT has excellent performance in real-time identification of innovation elements, network connection quality, sensor monitoring and AI vision monitoring, which provides solid technical support and scientific basis for the sustainable development of urban innovation networks. These contributions not only have positive significance for promoting the development of urban innovation ecosystem, but also provide useful experience for the research and development of similar systems in the future.

## 2 Methodology

### 2.1 Theoretical basis of innovative space design

Innovation space [12] is a complex network space and is formed based on continuous interaction, long-term cooperation, and real-time exchange of innovation elements in society. The network node in the innovation space is the innovation element, and the innovation network is the framework supporting the innovation space. Meanwhile, the innovation network is connected through the cooperative relationship between the innovation elements and plays a critical role in information exchange [13]. Additionally, the classification and coverage of innovation elements are constantly enriched and refined through an in-depth study of innovation elements and a summary of researchers' works. Specifically, innovative elements include higher education institutions, enterprises, research institutes, intermediary services, and local governments, policies, capital, materials, infrastructure, and ecological environment [14]. Here, the spatial entity of innovation elements is expressed abstractly to construct the support node of innovation space, while the relationship between spatial entities is expressed abstractly through the innovation network that can describe and distribute information and ensures effective transmission of information between innovation elements. Meanwhile, the innovation network is established through the cooperation relationship between different innovation elements, thus forming different types of cooperation networks, such as talent cooperation network, industry-university-research cooperation network, and scientific and technological achievements transformation network. The innovation network in this context is the distribution of points (innovation components) and lines (cooperation relationships) in space, which is an abstract representation of the cooperative interaction between innovation elements. Additionally, the idea's fundamental component is the innovation network. Table 1 presents the three dimensions that comprise the notion of the proposed innovation space.

**Table 1:** The connotation of innovation space.

Different dimensions	Main contents
Research objects of innovation space	It includes various abstract networks formed in space in innovation activities, such as knowledge dissemination and sharing networks, industry-university-research cooperation networks, production and transportation networks, and technological achievements transformation networks. These networks are established with innovation as the center.
Basic process of innovation space	It includes targeted and selective aggregation, diffusion, interaction, cooperation, and synergy based on spatial location.
Support of innovation space	It includes transportation corridors using policy planning and supporting media combining various information technologies, such as the Internet, innovation platforms, and electronic communications.

Secondly, the spatial structure of innovation space is explained. The concept of the point is introduced. The point is to express innovation elements abstractly [15]. In the innovation space, the innovation element can be expressed as a point after its size and the size of the existing regional space are ignored. The point is the fundamental building block of line and surface construction as well as the fundamental form of invention space. Afterward, the line can be defined. The line is an also abstract expression of innovation elements [16]. In the innovation space, when the distance between innovation elements is ignored, the cooperative relationship between elements can be abstracted as a line, and multiple intersecting lines form a network. The network is the backbone of innovation space and is the bridge of the flow of materials, information, and knowledge between elements. Additionally, the innovation network is crucial in the development of the innovation area. Finally, the concept of the plane is put forward. The plane refers to the basis of innovation and is the result of the quantification of the spatial distribution of different innovation elements [17]. The plane represents the geographic range of the influence of various innovation aspects and is the carrier of points and lines in the innovation space. According to regional economics, the genesis or emergence of economic nations serves as the foundation for the regional structuring of the economic flow rather than just being a regional concept [18]. Similar to this, innovation is built on the actions that various innovation elements inside the innovation area carry out.

Finally, the basic characteristics of innovation space are presented. Innovation space is an integrated space type, which is complementary to physical space, virtual space, and fluid space at different spatial levels. Thus, innovation space has virtual attributes of virtual space, material attributes of physical space, and fluid attributes of fluid space [19], as seen in Table 2.

**Table 2:** Basic characteristics of innovation Space.

Different attributes	Main contents
Material attribute	Innovation space is defined by its physical spatial geographic meaning, which is a distinct physical field of spatial geography. Innovation activities can be viewed as taking place in this space, which has limitations in terms of scale and area, for research and development, manufacturing, and achievement transformation.
Fluid attribute	Innovation space’ fluid attribute refers to the information flow of network nodes. Mobile information can vitalize the innovation network, generate and develop innovation space, and transform local physical space to network space, thereby making mobility the basic features of innovation space.
Virtual attributed	The virtual attribute of innovation space is the abstract expression of innovation space and is the cooperative relationships formed through the interaction between network elements with the support of computer technology and informatization means. In this way, the connection between a network and a spatial form is established and abstractly depicted.

### 2.2 Construction of urban innovation space system

**Fig. 1:**
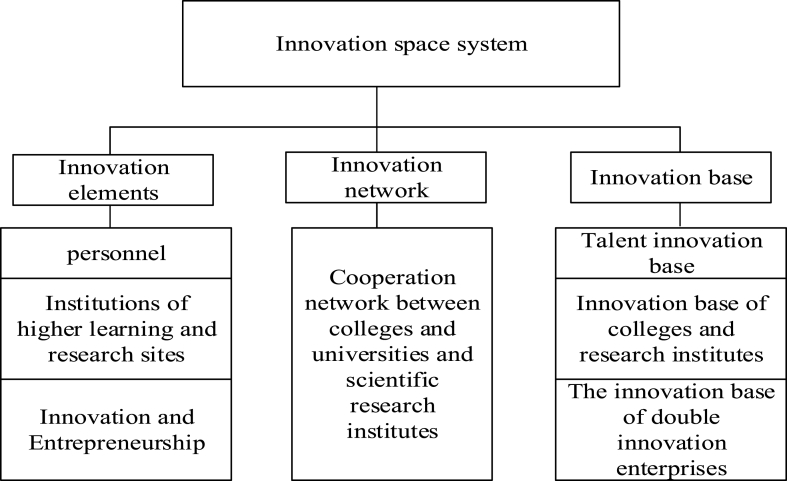
Urban innovation space system.

Using theoretical understanding, the urban innovation space system is constructed from innovation elements, innovation network, and innovation base. The specific structure is shown in Fig. 1.

In Fig. 1, innovation elements are the visual expression of innovation space [20], and the innovation base is the quantification of the spatial distribution of various innovation elements. Innovation network construction is the spatial expression of innovation cooperation networks formed by different innovation elements supported by different cooperation relationships. Finally, the innovation space system is constructed.

**Fig. 2:**
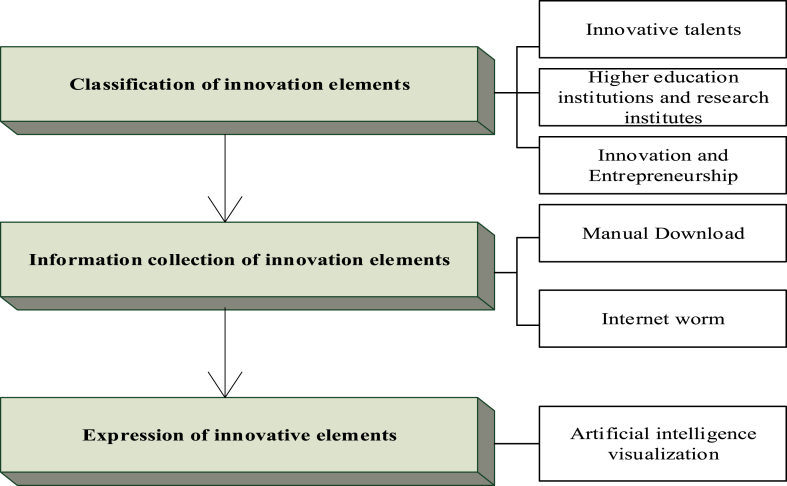
Construction process of innovation elements.

The first is the innovation elements. For different types of innovation elements, two key data acquisition technologies, Internet worm [21] and geocoding [22], based on the IoT are applied to realize data acquisition of innovation elements and AI visualization of spatial expression, as shown in Fig. 2.

**Fig. 3:**
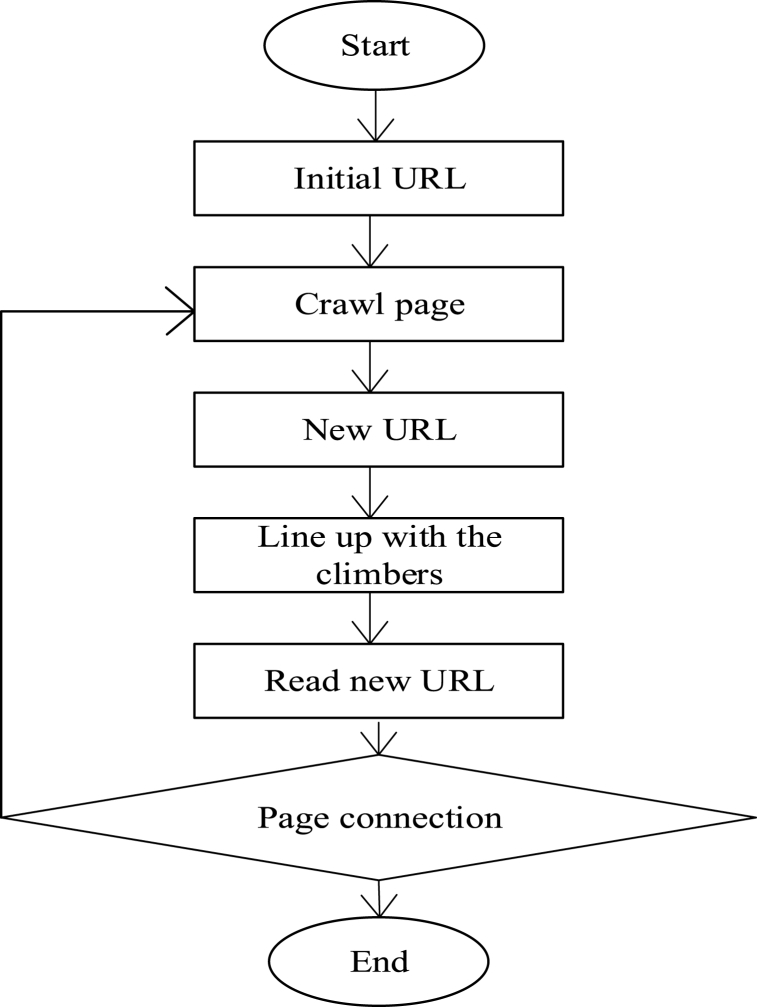
Technical steps of data collection using the Internet worm.

Innovative talents and innovative enterprises are the two categories into which the innovation ingredients fall [23]. During the data gathering process, some data can be manually downloaded from official websites. On the other hand, some websites do not provide a direct download path, and the data displayed on them is not readily available. When collecting enormous amounts of data, manual downloads have very low efficiency. To improve data gathering efficiency, Internet worm technology is presented. Here, the attributes of innovation elements can be automatically acquired through the Internet, thus improving the accuracy and effectiveness of key information acquisition and providing an effective mechanism for the collection of innovation elements. The technical route of the Internet worm technology in data collection is demonstrated in Fig. 3.

Subsequently, the download process of webpage data involves the IoT information fusion [24], address information arrangement for innovative elements collected by the Internet worm, including city, district, county, township, and house number. The standardized address structure includes the province, city, township, village or community, business area, street, and house number. These components of the address, together with the town, are essential. The innovation elements are expressed according to the structural information on their address, and the geospatial coordinates of innovation elements are indicated with the geocoding technology, thereby visualizing the innovation elements. While geocoding technology can connect text information containing spatial location with spatial information, integrate socio-economic information and spatial information, and provide positioning, analysis, visualization, and mapping functions of AI [25] for socio-economic information.

The next is the innovation network. The main innovation network studied here is the industry-university-research cooperation network, which is based on the cooperative joint data laboratory of entrepreneurship and innovation enterprises, higher education institutions, and research institutes, from which innovation elements are extracted.

**Fig. 4:**
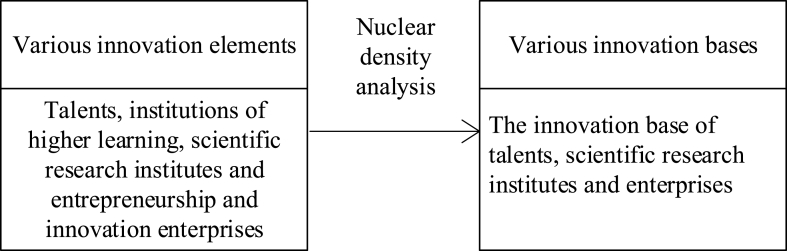
The implementation process of innovative base expression.

Lastly, the innovation base is the result of quantification of the spatial distribution of innovation elements, which support the development of innovation space. Fig. 4 depicts the proposed innovation base expression's implementation procedure.

Nuclear density analysis [26] is a common spatial feature density analysis method, which can calculate the density of input elements, point elements, and line elements in the field around each output pixel [27]. For the nuclear density analysis of point and line elements, a smooth plane is covered above each point or line, and the nuclear density at the position of the point or the line is the largest. The nuclear density, meanwhile, gradually falls as the distance from the point or line increases until it reaches zero at the location where the distance from the point or line is equal to the set search radius [28], as shown in Eq. (1).

(1) $$f\left(x\right)=\frac{1}{md}\sum_{a=1}^{m}\;\ker\;n\left(\frac{xa-x}{d}\right)$$

In Eq. (1), *d* represents the bandwidth, which controls the smoothness of nuclear density estimation and is selected according to the principle of Minimum Mean Square Error (MMSE). While kern () refers for the nuclear density function, *m* stands for the total number of observations.

### 2.3 Analysis tools, data sets, and parameter settings

(1) Here, the city of Chengdu, in Sichuan province in China is selected as the research base to analyze its innovation elements, innovation base, and innovation network. In terms of economic development, according to the statistical yearbook, in 2017, the city's GDP has exceeded 1388.9 billion RMB, and the per capita GDP has exceeded 85,000 RMB based on the resident population, so the economic operation is stable and good. In terms of innovative talents attraction, 120,000 full-time undergraduate and above young talents have settled in, and more than 30 people are introduced into the national thousand people plan. The city's high-tech enterprises have reached nearly 2,500, high-tech industry output value has exceeded 900 billion RMB. Meanwhile, more than 250 carriers of entrepreneurship and innovation have been built, more than 3000 projects have been organized and implemented, and more than 1.7 billion RMB has been invested in science and technology projects. Besides, more than 100,000 applications and more than 40,000 patents have been granted throughout the year.
(2) For the innovation network, the list of higher education institutions and research institutes released by the Chengdu Bureau of Statistics is manually downloaded. Afterward, the cooperation network data set is extracted for higher education institutions, research institutes, and cooperative units in Chengdu according to the data collation results. Then, the dataset is input into the ArcGIS Geographic Information System (ArcGIS) network model [29] to construct the innovation network and visualize the spatial expression of the industry-university-research cooperation network.
(3) For the innovation base, the nuclear density analysis is chosen to quantify the spatial distribution density of innovation elements. Nuclear density analysis is a common spatial analysis technology in Geographic Information System (GIS) [30]. The quantitative results directly reflect the spatial aggregation state of innovation elements, and the higher the spatial density of the innovation base is, the more supportive the innovation base is. The lowest spatial density of the innovation base is zero, which indicates that there is no innovation base in the corresponding spatial range. In nuclear density analysis, the grid size is 100 * 100 m. Finally, the search radius is selected to be 2 km through the comparison of the different search radius.

### 2.4 Feasibility analysis of research

This paper aims to apply AI and IoT technologies to urban innovation space system to promote urban information construction. This section analyzes the feasibility of this paper from many angles. First, it is necessary to evaluate the technical feasibility of AI and IoT technologies in urban innovative space systems. AI technology has proved its value in data analysis, decision support and automation in many fields. Meanwhile, IoT technology has been successfully applied to urban infrastructure monitoring and intelligent transportation systems. The continuous development and maturity of these two technologies make their application in urban innovation space system more feasible.

Chengdu, Sichuan Province is chosen as the research base because the city has rich resources and support to carry out this paper. Chengdu has first-class higher education institutions, research institutions and innovative enterprises. These resources provide a lot of research data and cooperation opportunities for the development of research. In addition, this paper has also obtained sufficient research funds and support to ensure the smooth progress of the research.

The research methods and plans are carefully designed to ensure that powerful research results can be obtained. In-depth data collection methods, including investigation, field observation and data analysis are be adopted to collect detailed information about Chengdu's innovation space. Advanced AI technology is also applied to visually analyze the data to reveal potential patterns and trends. These methods and plans ensure that the research is scientific and feasible.

## 3 Result and discussion

### 3.1 Analysis of innovation elements and innovation base

**Fig. 5:**
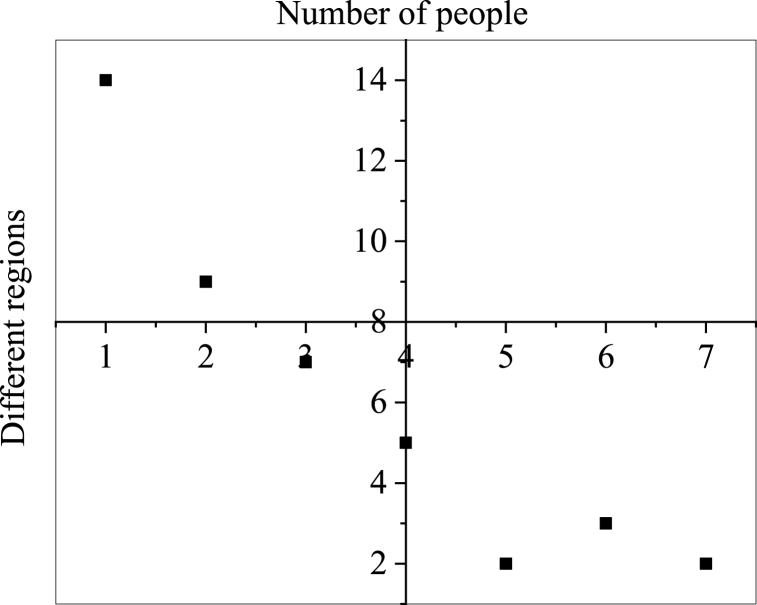
Visualization results of academician talent distribution in different regions of Chengdu (1: Jinjiang District 2: Wuhou District 3: Chenghua District 4: Jinniu District 5: Shuangliu District 6: High-tech District 7: Qingyang District).

The first is the talent analysis visualization. In this case, the talent spatial positioning is based on the academicians' working units. Fig. 5 illustrates how the spatial location of academicians in Chengdu is visualized to understand the talent distribution.

Fig. 5 implies that the academicians in Chengdu are only distributed in the central city. The Jinjiang District accommodates most academicians, with 14 members, the Wuhou District ranks the second, with 9 members, and Chenghua District ranks the third, with 7 members. Apparently, the first-tier city districts in terms of academicians number are in the central city, and the talent innovation base is proportional to the talent distribution. Therefore, there are large regional differences in the innovation bases in Chengdu, where some zero-innovation base exists.

**Fig. 6:**
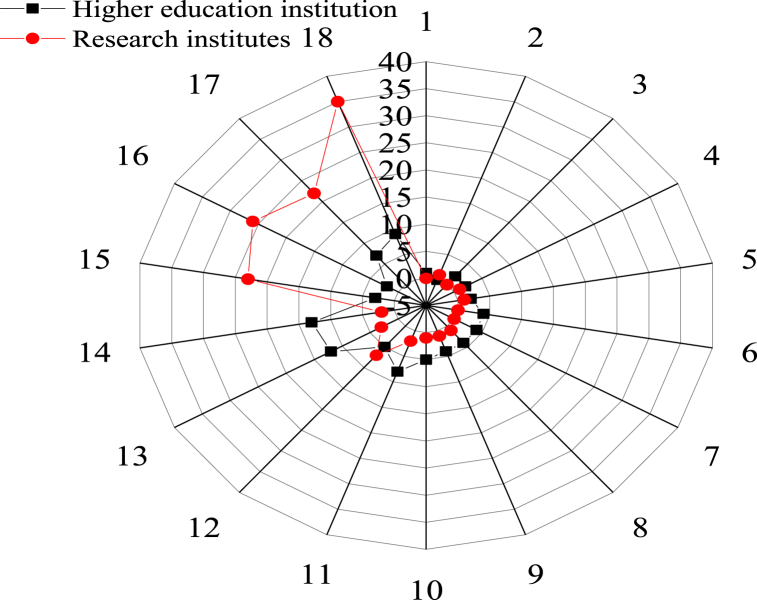
Higher education institution and research institutes (1: Pengzhou city 2: Qingbaijiang District 3: Dayi County 4: Chongzhou City 5: Xinjin County 6: Chenghua District 7: Jintang County 8: Tianfu New District 9: Xindu District 10: Dujiangyan City 11: Wenjiang District 12: Shuangliu District 13: Longquanyi District 14: Pidu district 15: Qingyang District 16: Jinjiang District 17: Jinniu District 18: Wuhou District).

Secondly, the higher education institutions and research institutes are analyzed through visualization, and the results are shown in Fig. 6.

Fig. 6 illustrates that most higher education institutions in Chengdu are concentrated in the central city and are sparsely distributed in the surrounding districts and counties. Fig. 6 suggests that higher education institutions and research institutes are distributed in 18 districts (cities) and counties in Chengdu. The Wuhou District is distributed with the largest number of higher education institutions and research institutes, totaling 44.

Thus, most higher education institutions in Chengdu are concentrated in the center of the city, with less distribution in the surrounding districts and counties. Meanwhile, higher education institutions and research institutes in Chengdu are distributed in 18 districts (cities) and counties, with the largest number in the Wuhou District, a total of 44. Jinniu District, Jinjiang District, and Qingyang District are also densely distributed. Wuhou District, which contains a total of 35 research institutes, is the district with the most, followed by Jinjiang District and Qingyang District. In terms of the number of higher education institutions, Pidu district has the largest number, followed by Longquanyi District and Wuhou District, respectively. Thus, the high-value areas of the base of higher education institutions and research institutes are concentrated in Wuhou District, Jinjiang District, and Qingyang District in the central urban area.

**Fig. 7:**
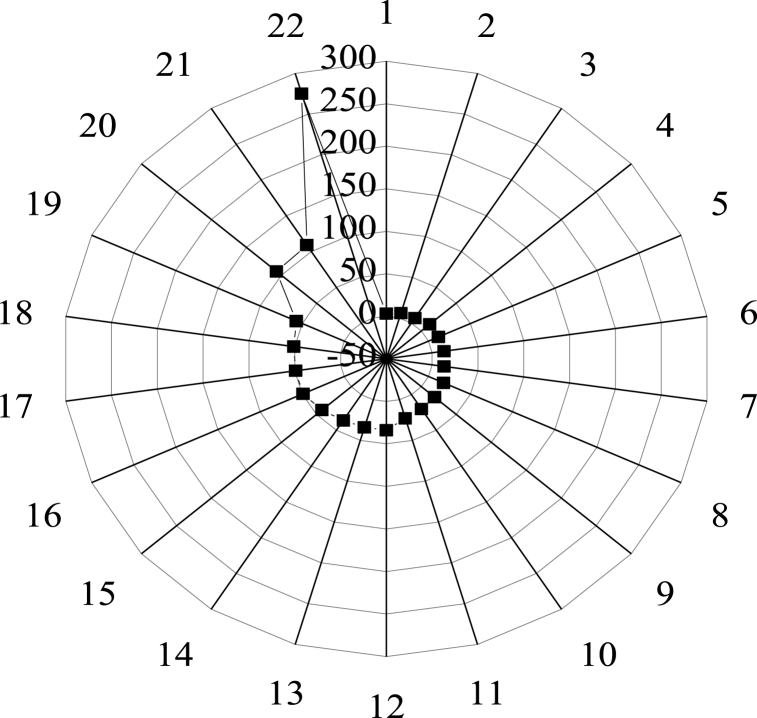
Statistical results of entrepreneurship and innovation enterprises in urban areas (1 Jianyang City 2: Pengzhou City 3: Pujiang County 4: Qionglai City 5: Dujiangyan City 6: Tianfu New District 7: Chongzhou city 8: Qingbaijiang District 9: Xinjin County 10: Jintang County 11: Dayi County 12: Wenjiang District 13: Chenghua District 14: Xindu District 15: Jinjiang District 16: Shuangliu District 17: Jinniu District 18: Longquanyi District 19: Qingyang District 20: Wuhou District 21: Pidu District 22: High-tech District).

Finally, the entrepreneurship and innovation enterprises are visually analyzed, and the results are shown in Fig. 7.

Fig. 7 reveals that entrepreneurship and innovation enterprises are distributed in all districts and counties of Chengdu. The High-tech District has the most number of entrepreneurship and innovation enterprises, with 275 in total, followed by 109 enterprises in Pidu District and 107 enterprises in Wuhou District, respectively. Thus, though entrepreneurship and innovation enterprises are distributed all across Chengdu, there is a significant difference in the number, and the high-value areas of innovation base are concentrated in five urban districts: Jinjiang District, Qingyang District, Jinniu District, Wuhou District, and Chenghua District.

### 3.2 Analysis of innovation network

**Fig. 8:**
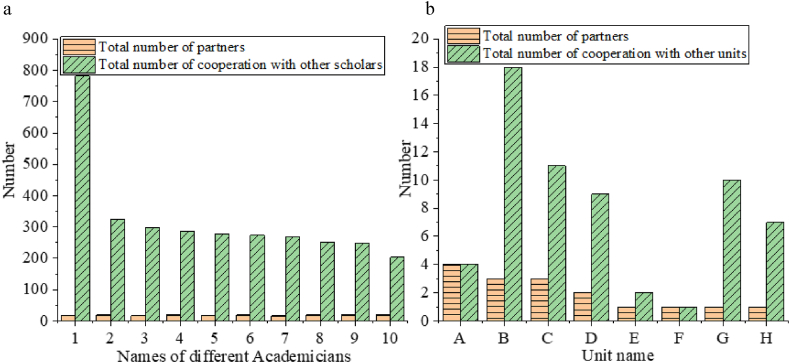
Visual analysis of characteristics of the top ten talent cooperation networks and cooperation networks of higher education institutions and research institutes (a: Statistics of top 10 talent cooperation networks 1: Tu Mingjing 2: Li Lemin 3: Cui Peng 4: Jiang Wenhan 5: Wei Yuquan 6: Zhai Wanming 7: Shi Bi 8: Qian Qingquan 9: Liu Shenggang 10: Li Yanrong b: Statistics of cooperation networks of higher education institutions and research institutes A: Southwest Institute of Electronics and Telecommunication Technology B: Sichuan University C: Chengdu University of Science and Technology D: University of Electronic Technology E: Chengdu Institute of Geology and Mineral Resources F: Southwest Jiaotong University G: Chengdu Institute of Biology, Chinese Academy of Sciences H: Chengdu Institute of Organic Chemistry, Chinese Academy of Sciences).

The talent cooperation network and the cooperation network of higher education institutions and research institutes are visually analyzed. The results are displayed in Fig. 8a and b.

Fig. 8 shows that in the top 10 talent cooperation networks, the first academician has cooperated with 18 partners and a total of 782 cooperations with other scholars. The academician ranking tenth has 19 partners and a total of 203 cooperations with other scholars. Eight different higher education institutions or research institutes have participated in the innovation network construction. The Southwest Institute of Electronics and Telecommunication Technology has the highest number of cooperation units, four times, while Sichuan University has the highest number of cooperations with other units, reaching 18 times.

### 3.3 Innovation space

**Fig. 9:**
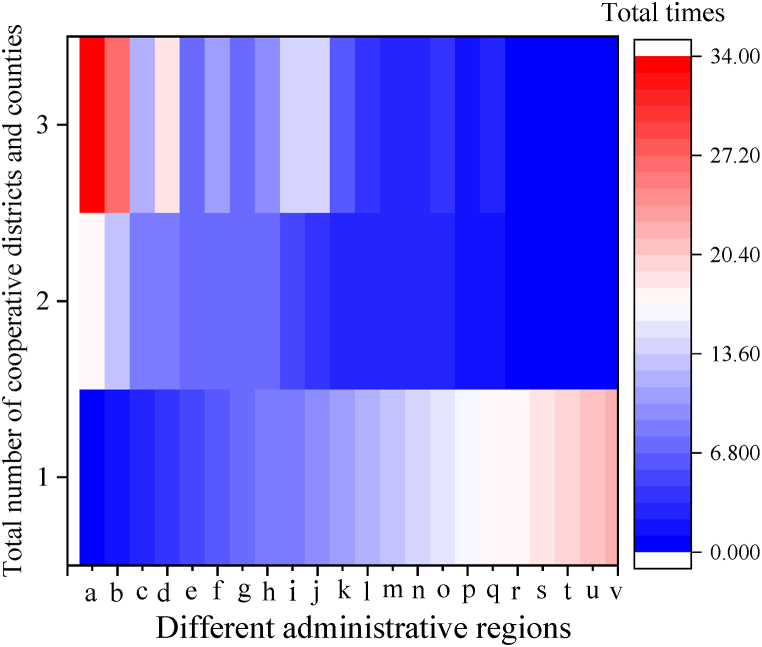
Typical regional characteristics of Chengdu innovation space system planning (a: Wuhou District b: Pidu District c: Jinniu District d: Chenghua District e: Qingyang District f: Shuangliu District g: Jinjiang District h: Wenjiang district i: Longquanyi District j: High-tech District k: Xindu District l: Dayi County m: Jintang County n: Xinjin County o: Dujiangyan City p: Qingbaijiang District q: Qionglai City r: Chongzhou city s: Pujiang County t: Tianfu New District u: Pengzhou City v: Jianyang City).

Innovation space is a comprehensive analysis of innovation base, innovation elements, and innovation network. The typical regional characteristics of Chengdu innovation space system planning are analyzed. The results are seen in Fig. 9.

Fig. 9 illustrates that the Wuhou District has 17 foreign cooperation zones and counties and a total of 34 cooperations, so the number of foreign cooperation zones and counties and the total amount of foreign cooperations rank first in Chengdu. The Pidu District has 13 foreign cooperation districts and counties and 27 times of foreign cooperations, ranking second in Chengdu. The Tianfu New District is the only area without a cooperation network in the central urban area, and Pengzhou and Jianyang in the suburban new cities have no innovation network of any kind.

Wuhou District has a high concentration of innovative talents and a solid foundation for innovation. Relying on Sichuan University and the Chinese Academy of Sciences, Wuhou District has established close cooperation with other districts and counties and has played a leading role in Chengdu's innovation space. In the ancient two rings, the Pidu District is the center of innovation collaboration between districts and counties and is crucial to the growth and development of the innovation network. As the key area of Chengdu's innovative city construction, the Tianfu New District has obvious spatial characteristics, only with massive distribution of innovative elements while without innovation networks. As a result, building an innovation network takes longer than investing in innovation elements, and sparse networks for innovation result in insufficient development of the innovation area. In the new suburban area, there are only a few innovation elements with a lack of innovation networks. Because there is a smaller labor force in the suburbs, there are fewer businesses and slower regional economic growth, which contributes to Chengdu's underdevelopment of the innovation industry.

### 3.4 Performance analysis results of innovative space system based on internet worm technology of IoT

The performance analysis results of the innovative space system based on Internet worm technology of the IoT are shown in Table 3. In general, the number of innovative elements is 150,000 and the number of innovative network connections is 2,500,000, which indicates that the system has considerable scale and complexity. The number of innovation bases is 30, which shows that the system has multiple innovation bases in the city. The sensor deployment density of the IoT is 500 sensors/km^2^, which indicates that the system has high-density sensing ability and is used to collect various data. The accuracy of AI vision analysis is 95 %, which shows that the system has high accuracy and reliability in processing image data. There are 275 innovative enterprises in Chengdu High-tech District, among which the distribution density of innovative enterprises is 30 enterprises/km^2^. The high number and distribution density show that the innovation activities in this region are relatively concentrated. There are 15 external partners in the High-tech District, which shows the cooperative relationship between the region and external institutions and enterprises. Meanwhile, there are eight universities participating in the urban innovation network, which shows that the system cooperates with academic institutions throughout the city. The total investment in the construction of innovation space network is 100 million RMB, which shows the financial investment involved in the construction of innovation network in this city. The analysis results show that the innovation space system based on Internet worm technology of IoT has extensive coverage and high technical support in urban innovation and cooperation.

**Table 3:** Performance analysis results of constructing innovative space system based on Internet worm technology of IoT.

Index	Total value	Subdivision data in Chengdu
Number of innovative elements	150,000	Area 1: 50,000
Number of innovative network connections	2,500,000	Area 2: 1,200,000
Number of innovation bases	30	Area 3: 10
Sensor deployment density of IoT	500 sensors/km^2^	Area 4: 700 sensors/km^2^
Accuracy of AI visual analysis	95 %	Area 5: 98 %
Number of enterprises in Chengdu high-tech District	275	High-tech District 1: 120
Distribution density of innovative enterprises in high-tech Districts	30 enterprises/km^2^	High-tech District 2: 40 enterprises/km^2^
Number of external partners in high-tech District	15	High-tech District 3: 15
Number of universities participating in urban innovation network	8	Area 6: 3
Investment in innovative space network construction	100 million RMB	Area 7: 50 million RMB

The monitoring results of AI technology on the generated innovation space system are shown in Table 4. It shows that the real-time identification rate of innovation elements is 98 %, which indicates that the system has high accuracy in real-time monitoring of new enterprises and projects, and can quickly and accurately identify emerging innovation elements. This has a positive impact on responding to market changes in time and supporting innovation activities. Secondly, the monitoring of innovation network connection quality shows that the communication quality is 92 %, which shows that the network connection is relatively stable and has good communication performance, which is helpful to ensure the effective information flow among the elements in the innovation space system and promote cooperation and innovation. In the aspect of sensor fault detection, the accuracy of sensor status monitoring in the IoT is 99 %, which means that the system can monitor the working status of sensors efficiently, find and solve potential problems in time, and ensure the reliability and stability of IoT devices. The coverage rate of AI visual monitoring reaches 96 %, which shows that the visual monitoring system has achieved good results in covering the city, which can effectively monitor the areas involved in the innovation space system and improve the perception range of various innovation activities. In the aspect of enterprise synergy monitoring, 87 % cooperation degree shows that there is synergy among innovative enterprises, but there may be room for further improvement to promote closer industrial cooperation and innovation. The monitoring of resource utilization rate of innovation base shows 94 %, which shows that the facility resources of innovation base are effectively utilized, which is helpful to improve the efficiency and quality of innovation activities. The monitoring result of external partner interaction is 90 %, which shows that the system has positive interaction with external enterprises and institutions, and provides strong support for the expansion of urban innovation network. The monitoring result of university participation is 95 %, which shows that universities have a high degree of participation in the urban innovation network, which is helpful to promote Industry-University-Research's cooperation and promote the application and innovation of scientific research results. The progress monitoring of innovation network construction shows 88 %, which indicates that innovation network construction has made some progress, but some aspects may still need to be paid attention to and strengthened to ensure the perfection and all-round development of the system. Finally, the result of security monitoring of innovative space network is 93 %, and the display system performs well in network and data security monitoring, which is helpful to prevent potential security risks. Generally speaking, these monitoring results reflect the good performance of the innovation space system based on the Internet worm technology of the IoT in many aspects, and provide a solid support and foundation for the innovation ecosystem of the city.

**Table 4:** Monitoring results of AI technology on the generated innovative space system.

Monitoring indicator	Specific monitoring content	Monitoring value
Real-time recognition rate of innovative elements	Real-time monitoring of start-ups and projects	98 %
Innovative network connection quality	Monitoring network communication quality	92 %
Sensor fault detection	Sensor condition monitoring of IoT	99 %
AI visual monitoring coverage rate	Coverage of visual monitoring system	96 %
Enterprise synergy monitoring	Cooperation degree of innovative enterprises	87 %
Monitoring of resource utilization rate of innovation base	Utilization of facilities and resources in innovation base	94 %
Monitoring of external partner interaction	Cooperation with external enterprises and institutions	90 %
Monitoring of university participation	Degree of university's participation in urban innovation network	95 %
Progress monitoring of innovative network construction	Implementation progress of innovation network construction	88 %
Security monitoring of innovative space network	Network and data security monitoring	93 %

## 4 Discussion

The research results of this paper have far-reaching influence on secure data transmission, biometric authentication, cognitive manufacturing system, architectural design and so on. By embedding AI technology into different fields, more efficient, innovative and safe system design can be realized. Annadurai et al. (2022) proposed a novel technology of intrusion detection in secure data transmission and biometric authentication system [31]. In this method, intruders were detected by collecting the biometric database of intelligent buildings based on IoT. In addition, the scholar believed that AI supports the development of the IoT and smart cities by creating devices that replicate intelligent behaviors and allow decision-making with little human intervention. Lazaroiu et al. (2022) believed that cognitive manufacturing system was based on sustainable product life cycle management, real-time production logistics based on the IoT and intelligent process planning assisted by deep learning, through optimizing value creation ability and decision-making algorithm based on AI [32]. Li et al. (2023) believed that AI played an auxiliary role in generating architectural intention and form. It mainly supported academic and working theoretical models and promotes technological innovation, thus improving the design efficiency of architectural design industry. AI-aided architectural design enables every designer to realize the freedom of design. With the help of AI, architectural design can complete the corresponding work more quickly and efficiently. In addition, with the help of AI technology, through the adjustment and optimization of keywords, AI automatically generates a number of architectural space design schemes. The auxiliary model of architectural space design is established, and the literature research is carried out through the analysis of AI model, intelligent auxiliary model of architectural space, semantic network and internal structure of architectural space [33]. This echoes the research of Li et al. (2023), who emphasized that AI can automatically generate a number of architectural space design schemes by adjusting and optimizing keywords. Through this intelligent aided model, this paper is committed to improving the efficiency and innovation of architectural space design. This also echoes the common understanding of scholars on the role of AI in their respective fields, and provides strong support for the application of intelligent technology in the future.

## 5 Conclusion

The theoretical study of the innovation space is imaginatively used in this case to the building of the innovation space system utilizing IoT-based Internet worm technology, and the produced innovation space system is visualized and analyzed using AI technology. The findings show that using IoT-based Internet worm technology, effective system development information can be gathered, and the data can be visually examined using AI technology. Chengdu's innovation space system presents obvious regional differences. The central city has richer innovation elements and denser innovation networks, while the suburbs are relatively backward. For the future urban planning and innovation space development, it is necessary to pay more attention to the balanced development among various regions of the city and promote the all-round promotion of innovation power. These research results provide powerful reference for urban planners, policy makers, and enterprises to better promote the construction of Chengdu's innovation ecosystem. When creating the urban innovation space system, it is important to take into account the distribution of talent inside the core city, as well as the characteristics of higher education institutions, research institutes, and entrepreneurship and innovation enterprises. On the other hand, Chengdu's uneven innovation space development is partly due to the absence of any form of innovation networks in many of the new outlying cities. However, shortcomings are also present. First, only three of these components, innovative skills, higher education and research institutions, and entrepreneurial enterprises, are studied, which means that there isn't much research on these aspects of innovation. Infrastructure and policy should also be considered in a more detailed analysis of the innovation components in the follow-up study.

## Data availability statement

The raw data supporting the conclusions of this article will be made available by the authors, without undue reservation.

## CRediT authorship contribution statement

**Yifang Gao:** Writing – original draft, Visualization, Software, Methodology, Formal analysis, Data curation, Conceptualization.

## Declaration of competing interest

The authors declare that they have no known competing financial interests or personal relationships that could have appeared to influence the work reported in this paper.
